# Rock climbing alters plant species composition, cover, and richness in Mediterranean limestone cliffs

**DOI:** 10.1371/journal.pone.0182414

**Published:** 2017-08-02

**Authors:** Juan Lorite, Fabio Serrano, Adrián Lorenzo, Eva M. Cañadas, Miguel Ballesteros, Julio Peñas

**Affiliations:** 1 Department of Botany. University of Granada. Granada. Spain; 2 Interuniversitary Institute for Earth System Research. Granada. Spain; Free University of Bozen/Bolzano, ITALY

## Abstract

Rock climbing is among the outdoor activities that have undergone the highest growth since the second half of the 20th century. As a result, cliff habitats, historically one of the least disturbed by human colonization worldwide, are facing more intense human pressure than ever before. However, there is little data on the impact of this activity in plant-communities, and such information is indispensable for adequate manager decision-making. The goal of this study was to determine the impact of rock climbing on plant communities in terms of cover, richness, and composition in relation to climbing intensity on typical Mediterranean limestone cliffs. Three rock-climbing sites were selected in the Baetic range (SE Spain), corresponding to qualitative categories of climbing frequentation: i)“low” (low frequentation with intermittent climbing), ii)“medium” (high frequentation without overcrowding), and iii) “high” (high frequentation with overcrowding). Within each site, we selected climbing routes and adjacent areas free of climbing, then we carried out a photoplot-based sampling by rappelling. We analysed the images to calculate: richness, species cover, and total cover. This study shows that rock climbing negatively affected the cliff plant community at all three study sites. A significant decrease in plant cover, species richness and a shift in the community composition were recorded for climbed areas, the cover being the variable most sensitive to rock climbing. Impact observed proved to be related to the frequentation level. Low-frequentation sites, with usually more specialized climbers, underwent relatively mild damages, whereas at high frequentation sites the impact was severe and the conservation of the species, especially rare ones, became jeopardized. Our study is the first one available to investigate climbing impact on plant communities in Mediterranean areas, but more research on the impact of rock climbing is needed to assess the regulation of this activity. Regarding management guidelines, we propose a management guideline protocol to evaluate climbing routes and design: i) “Sites free of climbing”, ii) “Strictly regulated climbing routes”, iii) “Mildly regulated climbing routes”, or iv) “Free climbing routes”.

## Introduction

Since the 1950s, recreation and tourism have expanded exponentially in natural areas worldwide, both in the number of practitioners as well as in the number of activities practised [1]. This explosion in visits has caused disturbances and, accordingly, environmental impact, which in turn raises concerns as to whether recreation and tourism activities in protected areas can be sustainably managed [2]. Consequently, there are growing efforts to understand the ecological effects of this trend. In fact, a specialized field of study called Recreation Ecology has emerged to examine the impact of outdoor recreation and nature-based tourism in natural or semi-natural environments [3]. As a result, a range of direct and indirect consequences of recreational activities has been documented mostly in North America, Europe, and Australia [4]. However, most of the repercussions are far from being fully understood [2], and therefore more research is needed to ensure the sustainable management of these activities.

Rock climbing is one of the outdoor activities that has most increased over the second half of the 20th century [5,6,7]. For example, climbing increased 8% in the USA between 1980 and 1984, 12% between 1985 and 1990 [8], and 10% between 1999 and 2009. This number is expected to rise 50% by the year 2050 [1]. As a result, cliff habitats, previously regarded as one of the least anthropogenically disturbed worldwide [9], are facing greater human pressure than ever before [10]. At the same time, the relative inaccessibility of these habitats has largely prevented biologists from performing research, resulting in a relative scarcity of information on the impact of rock climbing until today [4, 11], leaving a gap in knowledge needed for adequate manager decision making to this regard [11].

Research in Germany [12,13,7], Switzerland [14,6,15], Canada [9, 16,17,5], and the United States [18,19,20] has helped evaluations on the ecological effects of rock climbing, focusing on changes in the structure and composition of cliff-face vegetation. Almost all studies on the impact of rock climbing have found consistent negative effects on plant species (see [11] for a review), including i) reduction of plant cover; ii) decreasing of species richness; iii) extinction of species sensitive to disturbances and of specialist species adapted to these extreme habitats; and iv) expansion of ruderal-colonizer or alien species [5,7]. These changes in plant community may also alter soil properties (i.e. soil texture, fertility, and productivity) or microclimate, resulting in a cliff area more prone to disturbances [21]. These mechanical effects begin with the establishment of new climbing routes, which results in trampling, or pulling most of the plants (commonly referred to as “cleaning” or “gardening”; [21, 22]). Then, trees and shrubs are used as rappel or belay anchors, causing damages at the medium term [9]. Rock climbing also mechanically damages plants by partially or fully eliminating them [6,17]. In the absence of disturbances, these plant species are usually long-lived because of low mortality rates linked to a harsh environment but with low disturbances [23] Therefore, these areas may undergo arrested recovery when disturbed.

This pressure is especially harmful for rare and/or endemic species that are particularly frequent in these habitats. This has played a key role in the loss of biodiversity at the local and regional scale [24].

Within the Mediterranean hotspot [25] cliff faces have acted as anthropogenic and environmental refuges, resulting in unique habitats with a high number of rare and endemic species [26,27], particularly on limestone cliffs. Polunin (see [26] p. 44) stated that *“each gorge or cliff will have its own collection of species and many rarities are only to be found in such habitats”*. Scientifically, these habitats have proven highly useful in addressing a great number of ecophysiological [28] and evolutionary issues [29], thereby constituting a priority for biodiversity conservation [30].

However, the characteristics of cliff-face habitats, as mentioned above, make it difficult to apply sampling and monitoring methods, usually developed for more accessible areas [23]. Therefore, the surge in climbing activity is not matched by proportional monitoring and evaluation of the impact [31]. In fact, no research is available on the ways in which climbing affects plant communities in the Mediterranean hotspot. Within this area, the Baetic range (south-eastern Spain) has high plant diversity, not only in flora as a whole [32, 33], but also in chasmophytes (i.e. plants that grow in rock crevices) [27], making the area ideal to study the effect of rock climbing.

In Spain, as in other Mediterranean countries, the number of climbers has risen some 25% in the last decade, with ca. 99,000 climbers. The south of the country being particularly attractive to climbing activities for climatic reasons (i.e. few rainy days and mild winter temperatures) (www.fedme.es). In many protected areas, managers are facing increasing pressure in the existing climbing routes and a growing demand for establishing new ones. Managers are making contrasting decisions, from closure of existing routes, to the approval and even the promotion of the opening new ones. All these decisions are made without proper evaluation to support them [34], and have been contested by climbers. Therefore, it is crucial to generate scientifically sound information that facilitates and provides criteria for decision-making processes among managers. The goal of this study was to determine the impact of rock-climbing on the cover, richness, and composition of plant communities growing on typical Mediterranean limestone cliffs.

## Material and methods

### Study area

We selected three rock-climbing sites: Salto de la Cabra (“Cabra” hereafter), Alfacar and Cahorros (see Table 1 for further details) located in the Baetic range (SE Spain), a recognized biodiversity hotspot within the Mediterranean hotspot [33]. Selected areas were located between 650 and 1000 m asl. The climate type is continental Mediterranean, with relatively cold winters, hot summers, and four months of water deficit. The mean annual temperature ranged between 14.1°C and 16.9°C, with an average monthly minimum temperature in January (5.6–11.8°C) and a maximum in August (22.1–24.8°C). Annual rainfall averages ranged from 479 to 760 mm, occurring mainly in winter. The sites occupy part of the Baetic-Rif geological complex, the cliffs studied being composed mainly of limestone and dolomite [35].

**Table 1:** Main features of the study sites.

Site	Coordinates	Altitude (m asl)	Mean T (ºC)	Min (ºC)	Max (ºC)	Average rainfall (mm)	Protected area type	Climbing intensity
**Cabra**	37º41’46”N 3º45’46”O	650	16.9	11,8	22,1	760	Periurban Park	Low
**Alfacar**	37°15'22"N 3°33'40"O	1200	14.1	5,6	24,5	526	Natural Park	Medium
**Cahorros**	37°7'39"N 3°31'3"O	1000	14.8	6.4	24.8	479	Natural Park	High

Plant communities living on cliff-faces in the study area are typically composed of plant species of Mediterranean origin with some rare and/or endemic cases, the most abundant and/or typical being *Sarcocapnos pulcherrima*, *Teucrium rotundifolium*, *Chaenorhinum villosum*, *Chiliadenus glutinosus*, *Ceterach officinarum*, *Polygala rupestris*, *Sedum dasyphyllum* and *Phagnalon rupestre* [36].

### Sampling design and data collection

In the absence of detailed data on the number of climbers per site, the frequentation was assigned using expert judgement (according to [37] and A.J. Herrera, pers. com.), resulting in three qualitative categories: i) “low” (low frequentation with intermittent activity) for Cabra, ii) “medium” (high frequentation without overcrowding) for Alfacar, and iii) “high” (high frequentation with overcrowding) for Cahorros (see Table 1).

Within each of the three sites, we selected climbing routes and adjacent areas with the same conditions at least 5 m away from the climbing routes and without any sign of climbing activity. We sampled the transects matched in pairs (climbed/unclimbed) in order to avoid differences in exposure, inclination, etc. The selected climbing routes sampled here represent medium climbing-difficulty standards (lower than 6b in the French scale; UIAA: http://www.theuiaa.org), and style (i.e. sport climbs with fixed-bolt protection). These are the most commonly used for rock climbing at the sites, and therefore supporting the greatest impact [9,6]. No permission was required because sampling was performed in non-regulated climbing routes and we did not collected specimens of protected plant species.

We established 34 transects with 410 quadrats (206 unclimbed, 204 climbed), 110 for Cabra, 120 for Alfacar and 180 for Cahorros (see Table 1). We sampled each transect by rappelling. The climbing rope was fixed to safe elements such as rocks, or trees, and the sampling was made in the descent (partially adapted from [23]). We carried out a photoplot-based sampling [38], taking pictures with a digital camera of 0,25 m^2^ quadrats, placed every 2 m along the transect, resulting in 10–15 quadrats (= photos) per transect (adapted from [20]).

### Data analysis

We analysed the images with ImageJ 1.47v software [39]. In each photograph we marked the orthogonal projection of each individual plant, for each plant species separately. The following variables were calculated: i) richness, as the number of different species encountered per quadrat, ii) species cover, as the percentage of the total surface covered by a given species, and iii) total cover, as the sum of all species cover per quadrat. We identified plant species in the photographs, collecting some specimens in order to identify them in the laboratory under a binocular microscope when necessary. For identification and classification, we followed *Flora of Eastern Andalusia* [40].

### Statistical analysis

All statistical analyses were implemented in R version 3.3.0 [41]. First, we explored differences in cover and richness by use (climbed/unclimbed) and by site, fitting generalized linear models (GLMs) with a Poisson error and log-link function distribution. Multiple comparisons were performed using the R “multcomp” package [42]. For the 14 most abundant species, GLMs with a Poisson error distribution were also fitted to explore the effect of climbing on cover by species and, n^2^ to test the differences in presence (i.e. % of sampling plots where the species is present).

Afterwards, to test differences in cover and richness among sites (Cabra, Alfacar and Cahorros) combined with the use (i.e. climbed vs. unclimbed), we fitted Generalized Linear Mixed Models (GLMMs) using “glmer” function (R “lme4” package; [43], with a Poisson error distribution. These GLMMs enable us to deal with the different levels of pseudoreplication inherent to the sampling. Therefore, we nested quadrats in plot and site as random factors in the models. In the final models, we removed the interaction between sites (Cabra/Alfacar/Cahorros) and use (climbed/unclimbed) that were not significant either for cover or for richness. Afterwards, we calculated the variance explained by the models (as R^2^) using “r.squaredGLMM” function [44] implemented in MuMIn R package [45].

We also explored changes in the composition of plant communities by means of multivariate analyses. First, we explored the influence of the categorical factors (site and use) over the matrix of species per transect by means of a permutational multivariate analysis of variance (permanova), performed with the “adonis” function in R package vegan 2.0–10 [46]. Then, we used Canonical Correspondence Analysis (CCA) to examine whether the composition of plant species differed between climbed and unclimbed areas and the relationship with the plant cover and richness. For CCA analysis, the data were averaged per transect and then transformed using Wisconsin double standardization (i.e. species abundance are first standardized by maxima /by species maximum, and then samples by total, and by convention multiplied by 100), which improves the gradient-detection ability, especially in zero-inflated matrices, as typically occurs in rock-crevice sampling [11]. Also, for this analysis, the factor “site” was transformed into three variables (one per site) taking 0/1 values. Finally, we fitted environmental variables, both categorical and continuous, onto the CCA ordination using the function “envfit” in the R package vegan.

## Results

A total of 391 individual plants of 34 species were recorded in the 410 quadrats sampled (113 individuals from 20 species in the climbed quadrats and 278 individuals from 25 species for the unclimbed ones). The most frequent species were chasmophytes such as *Teucrium rotundifolium*, *Chiliadenus glutionosus*, *Sedum dasyphyllum* and *Chaenorrhinum villosum*.

**Fig 1:**
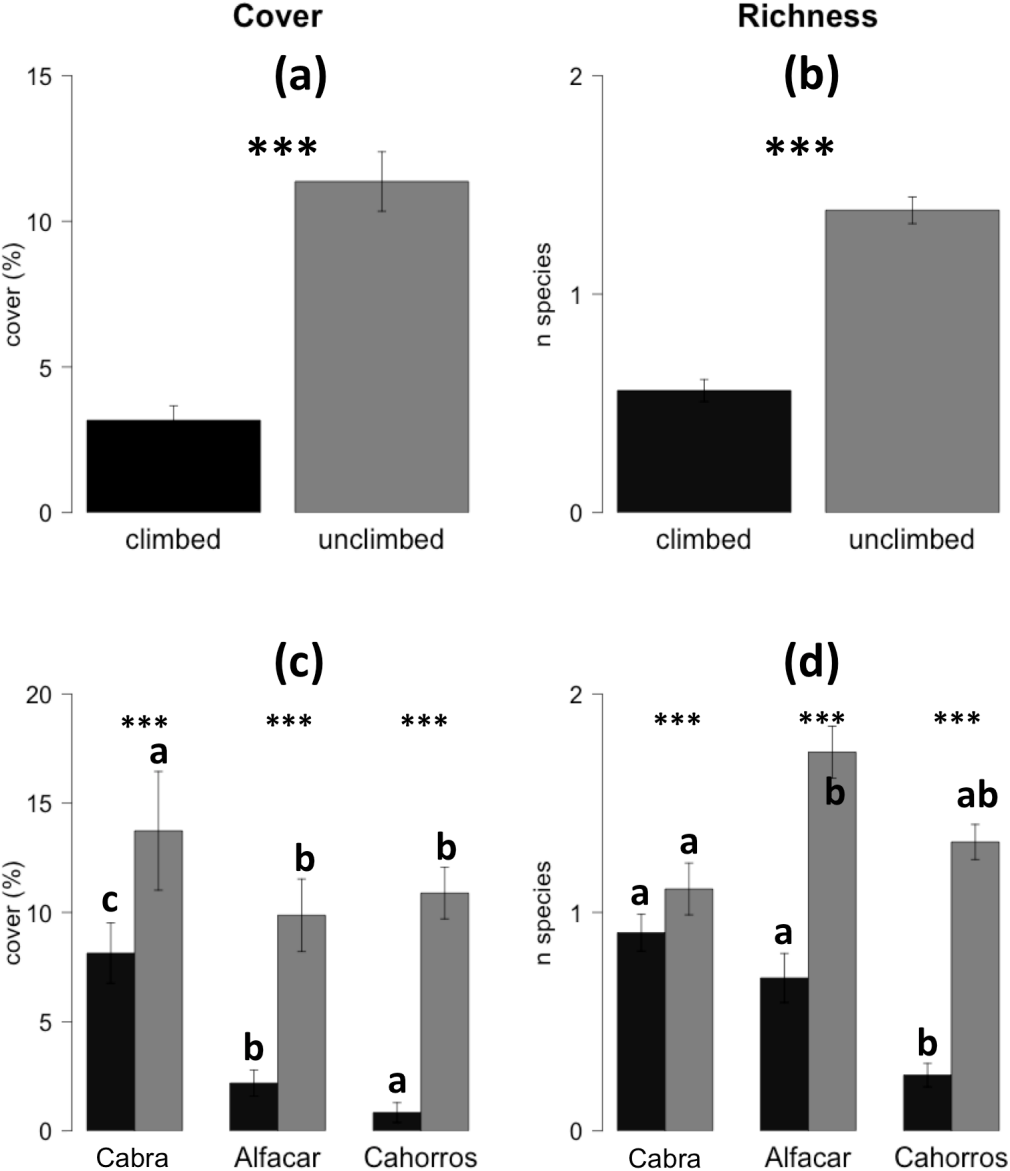
**Mean (± SE of the mean) of climbed vs. unclimbed areas of a) plant cover b) richness, c) cover per area and d) Richness per area.** Significant codes above the bars indicate the p-values obtained after the GLMs as follows: n.s. (not significant) p>0.05; * p< 0.05; **p< 0.01; ***p< 0.001. Different letters in c) and d) indicate significant differences across the sites with the same use (climbed or unclimbed) at p<0.05 in the post hoc Tukey tests.

The overall cover of the quadrats was low (7.29±0.61%), being significantly higher in unclimbed areas in comparison with climbed ones (11.36±1.02% vs. 3.17±0.49; p<0.000; Fig 1A). Richness showed the same pattern, with 0.97±0.04 species per quadrat (unclimbed = 1.38±0.06 vs. climbed = 0.72±0.05; p<0.000) (See Fig 1B). Within the three studied sites, both cover and richness were significantly higher in all cases for unclimbed areas (Fig 1C and 1D). Going one step further, we found that for unclimbed areas, cover was significantly higher in Cabra compared with the other two sites (Fig 1C), while richness was significantly higher in Alfacar than in Cabra, Cahorros having intermediate richness (without significant differences with the other two sites). In the climbed sites, both cover and richness drastically decreased, with significant differences related to the climbing frequentation level, this being lower in Cabra, higher in Cahorros, and intermediate in Alfacar.

GLMMs revealed that cover differed depending on the use (climbed vs. unclimbed) while no differences were found among sites (Table 2). For richness, unclimbed areas also registered significant differences. Moreover, climbed areas in Cabra differed with respect to the other sites, indicating that richness was better preserved in these routes with lower climbing frequentation. Cahorros, with a high climbing intensity showed marginally significant differences, richness being negatively influenced (Estimate = -0.41686) by this high frequentation.

**Table 2:** Generalized Linear Mixed Models (GLMMs) evaluating the effect of climbing over plant cover and plant richness.

**Response = cover**		**Estimate**	**Std. Error**	**z value**	**Pr(>|z|)**
	(Intercept)	0.4964	0.5459	0.909	0.363
	Medium (Alfacar)	-0.7890	0.5994	-1.316	0.188
	High (Cahorros)	-1.3812	0.5785	-2.387	0.017 *
	Unclimbed	2.3460	0.3812	6.155	7.52e-10 ***
	^a^ Model R^2^ = 0.4182				
**Response = richness**		**Estimate**	**Std. Error**	**z value**	**Pr(>|z|)**
	(Intercept)	-0.57703	0.19911	-2.898	0.00375 **
	Medium (Alfacar)	0.08209	0.21888	0.375	0.70761
	High (Cahorros)	-0.41688	0.21482	-1.941	0.05231
	Unclimbed	1.07216	0.16004	6.699	2.09e-11 ***
	^a^ Model R^2^ = 0.2732				

^**a**^ R^2^ of the GLMM model.

Significant codes for p-values obtained after the GLMMs

^.^ p< 0.1

* p< 0.05

**p< 0.01

***p< 0.001

The permanova analysis indicated that Site exerted the greatest influence over the species composition (F = 2.7180; R^2^ = 0.13843; p = 0.002), but also climbing had a significant influence (F = 3.9305; R^2^ = 0.10009; p = 0.001), and there was a significant interaction between these two factors (F = 1.4517; R^2^ = 0.07394; p = 0.044; see S1 Table for further details). This suggests that species composition changes among sites, and are being influenced by their use (climbed vs. unclimbed), consistent with the results shown in Fig 1.

**Fig 2:**
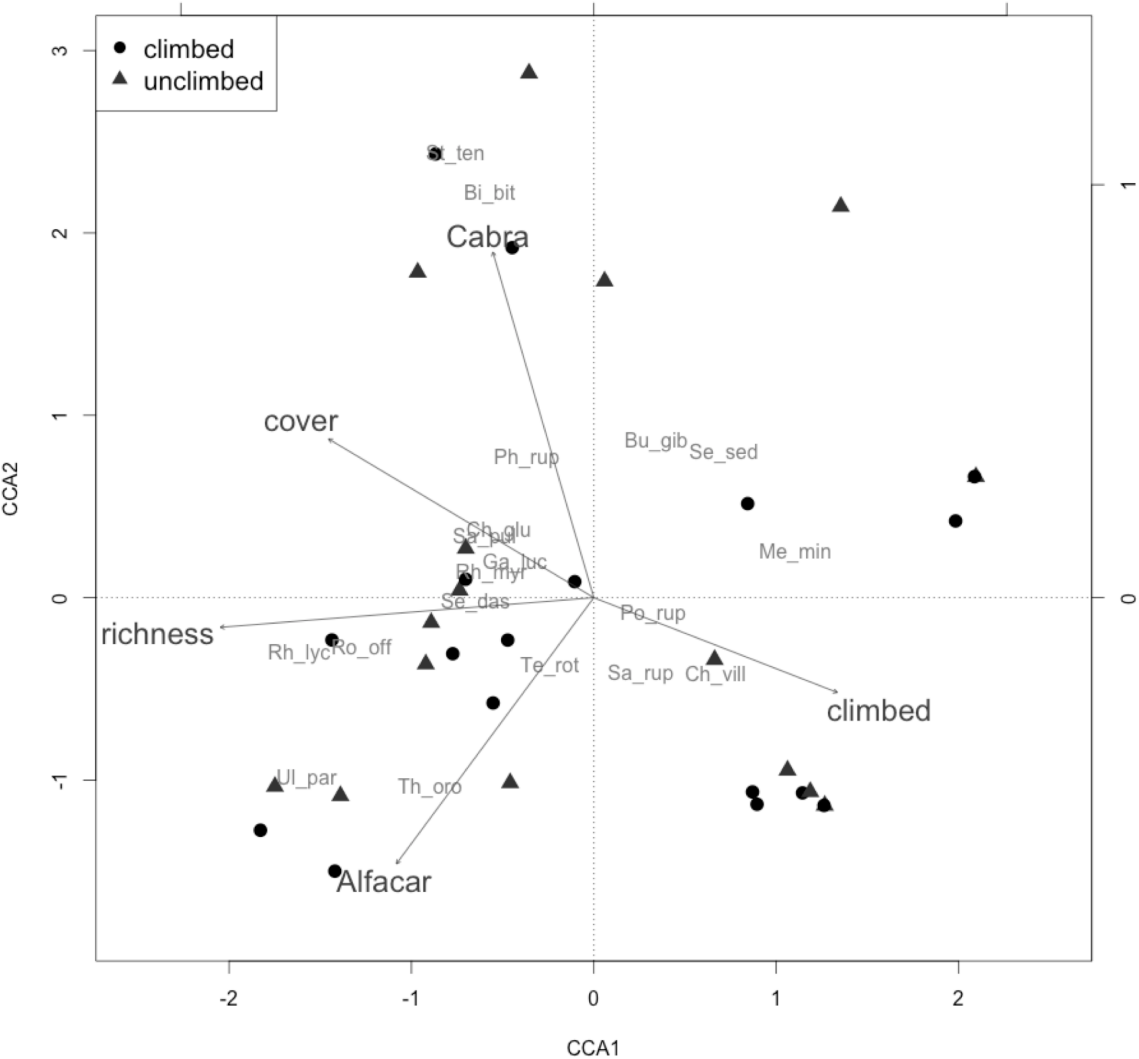
Biplot showing the result of CCA (Variance explained: CCA1 = 33.05%; CCA2 = 23.26%). Transects climbed marked with black circles and unclimbed by triangles. Species (in grey): Bi_bit = *Bituminaria bituminosa*, Bu_gib = *Bupleurum gibraltaricum*, Ga_luc = *Galium lucidum*, Ch_glu = *Chiliadenus glutinosus*, La_mar = *Lavatera maritima*, Me_min = *Melica minuta*, Ph_rup = *Phagnalon rupestre*, Po_rup = *Polygala rupestris*, Rh_lyc = *Rhamnus lycioides*, Rh_myr = *Rhamnus myrtifolius*, Sa_pul = *Sarcocapnos pulcherrima*, Se_das = *Sedum dasyphyllum*, Se_sed = *Sedum sediforme*, St_ten = *Stipa tenacissima*, Ch_vill = *Chaenorrinum villosum*, Ul_par = *Ulex parviflorus*, Ro_off = *Rosmarinus officinalis*, Sa_rup = *Sanguisorba rupicola*, Te_rot = *Teucrium rotundifolium*, Th_oro = *Thymus orospedanus*, He_hel = *Hedera helix*, Ce_off = *Ceterach officinarum*, So_ten = *Sonchus tenerrimus*, Me_sat = *Medicago sativa*, Si_bor = *Silene boryi*.

Canonical ordination was performed based on 19 species that appeared at least in 5% of transects. The first two axes of the analysis accounted for 56.31% of the variance (see Fig 2 and S2 Table). Composition varied among the three study sites (climbing intensity). Cover and richness were both positively correlated between them and negatively correlated with climbed areas. Most of the species appeared in association with the variables cover and richness and also with Cabra and Alfacar sites and negatively associated with climbed areas. This result was especially noteworthy for species with larger size, such as *Rhamnus lycioides*, *Ulex parviflorus* or *Rosmarinus officinalis*, which are usually removed during climbing routes conditioning, and/or damaged by climbing, especially in route with high frequentation.

Differences in cover per species, despite being low, were significant for 13 of the 14 most abundant species (see Table 3), the greatest being in unclimbed areas. Exceptions were *Chaenorhinum villosum*, which was significantly higher in climbed areas, and *Sanguisorba rupicola*, which showed no significant differences.

**Table 3:** Effect of rock climbing on the main species in terms of cover (%) and presence (%).

	cover (%)^a^			presence (%)^b^		
Species	Unclimbed^c^ (n = 206) ^d^	Climbed^c^ (n = 204) ^d^	Deviance^e^^,^^g^	Unclimbed^c^ (n = 206) ^d^	Climbed^c^ (n = 204) ^d^	χ^2^^f^^,^^g^
*Bituminaria bituminosa*	0.11±0.06	0.05±0.04	4.32 *	1.94	0.98	0.32 n.s.
*Chaenorhinum villosum*	0.10±0.03	0.19±0.06	5.67 *	10.68	9.80	0.04 n.s.
*Chiliadenus glutinosus*	1.10±0.35	0.42±0.18	65.19 ***	16.50	3.92	7.75 **
*Melica minuta*	0.49±0.15	0.01±0.00	131.73 ***	7.77	0.98	5.27 *
*Phagnalon rupestre*	0.11±0.06	0.01±0.00	24.42 **	1.94	0.49	0.86 n.s.
*Rhamnus myrtifolius*	2.35±0.73	0.63±0.24	216.74 ***	9.71	4.41	1.99 n.s.
*Rosmarinus officinalis*	0.62±0.35	0.01±0.00	173.7 ***	5.34	0.49	4.04 *
*Sarcocapnos pulcherrima*	1.00±0.33	0.28±0.10	89.33 ***	9.22	4.90	1.32 n.s.
*Sedum dasyphyllum*	0.30±0.09	0.15±0.05	11.02 **	12.62	7.84	1.12 n.s.
*Sedum sediforme*	0.18±0.08	0.09±0.05	5.94 *	3.40	2.94	0.03 n.s.
*Stipa tenacissima*	0.55±0.25	0.23±0.18	27.29 ***	2.91	0.98	0.96 n.s.
*Sanguisorba rupicola*	0.07±0.04	0.06±0.05	0.23 n.s.	2.43	1.96	0.05 n.s.
*Teucrium rotundifolium*	2.37±0.34	0.16±0.05	469.21***	34.95	7.35	18.01***
*Ulex parviflorus*	0.15±0.10	0.01±0.00	43.88 ***	2.43	0.00	2.43 n.s.

^**a**^ GLMs performed for cover comparing unclimbed vs. climbed areas.

^**b**^ For presence (%) differences were assessed by mean of χ^2^ test.

^**c**^ Mean values ± S.E. are shown

^**d**^ n indicates the total number of 0,25 m^2^ sampling plots.

^**e**^ Deviance values obtained from GLMs

^**f**^ Values from χ^2^ test

^**g**^ Significant codes for p-values obtained: n.s. (not significant) p>0.05

* p< 0.05

**p< 0.01

***p< 0.001.

Regarding species presence (i.e. % of sampling plots containing the species), significant differences between climbed and unclimbed areas, were found only for *Chiliadenus glutinosus*, *Melica minuta*, *Rosmarinus officinalis*, and *Teucrium rotundifolium*. The presence of these species was consistently higher in unclimbed areas, generally showing a sharp reduction in climbed areas (see Table 3).

## Discussion

The present study shows that rock climbing negatively affected the cliff plant community at the three study sites. A significant loss of plant cover, species richness, and a shift in the community composition were recorded for climbed areas. Similar climbing-related changes in vegetation have been reported in other studies [47,12,19,18,20,9,5,6,11]. In the present study, these changes were consistently related to the level of climbing frequentation, being especially severe for Cahorros, the site with highest frequentation and greatest overcrowding. The same pattern for overcrowded areas has been observed by other authors [18].

Cover was the most sensitive response variable to rock climbing. Damages were due to route-conditioning (via eliminating taller plants and creating additional hand and foot holds) as well as for the climbing activity itself (i.e. mechanical damage) [9,18,6].

Richness decreased in overcrowded areas following the same pattern as for cover. However, the pattern was not so sharp. In fact, the plant individuals remained even in overcrowded areas, but with a marked decrease in cover. In this sense, microsites formed by rock irregularities can play an important role in terms of the resilience of the plant community [16], constituting safe sites for plant survival. However, overcrowding can drastically reduce these safe microsites [16]. In this sense, in the areas with threatened species, restrictions (in terms of climbing period or climbing frequentation) or even closure of areas might be necessary in order to guarantee their conservation [10,48].

The plant community at sites with highly frequent climbing also underwent major changes. In particular, hardwood or spiny shrubs and dwarf shrubs are often removed in the preparation of climbing routes, while dwarf sub-shrubs often remain [9]. Also, species that can be easily harmed (i.e. herbaceous or weak stems) are more prone to disappear. In particular, *Chiliadenus glutinosus*, *Sarcocapnos pulcherrima* or *Teucrium rotundifolium*, show pronounced differences, both in abundance and cover in comparison with other less fragile dwarf shrubs, as also pointed out in other research [6].

The most significant changes in the community composition were observed in terms of frequentation. In particular we detected a decline in several rare and endangered species with climbing activity, this being particularly clear for *Sarcocapnos pulcherrima*. The same was found by other authors [5,6], affecting also their population genetic structure [7]. However, as opposed to other researchers, we did not detect any replacement by ruderal-colonizers or alien species [5]. Contrasting results concerning changes in the grass and colonizer abundance have been reported by different authors. For example, Farris [20] and McMillan & Larson [5] found greater grass abundance, while Nuzzo [19] and Camp & Knight [18] reported lower grass abundance due to climbing activities. Later, other authors suggested that the abundance of grasses increased under low climbing intensity, but decreased at very high climbing intensity [6]. These differences with previously published work might be caused by variation in floristic pools and environmental conditions across studies. In our study can also be inherent to Mediterranean rock crevices.

The impact observed was related to the climbing-frequency level. At sites with low levels, and usually more specialized climbers (with usually more demanding routes and/or difficult access), the damages were relatively low. In contrast, at overcrowded sites such as Cahorros, the impact became severe, jeopardizing the conservation of species, especially rare ones.

Our study is the first one available devoted to climbing impact on plant communities in Mediterranean areas. Much research is needed to shed light on this issue. Our study is restricted to beginner–intermediate climb difficulties, as most of previous studies (up to 6b in French scale or 5.9 YDS; e.g. [5,9]. Some authors have noted the strong influence of microtopography, in relation with the intensity of the use and with the potential damage to plants [9,11]. Therefore, the potential damage in the more technical routes is presumably lower. Moreover, we have not considered annual species in this study, because sampling was partly performed late in growing season, and this could introduce artificial differences/sampling artefacts between early and late sampled areas [20]. However, we presumed that these annual species are less abundant in the Mediterranean cliff habitats that are subject to comparatively harsher environmental conditions.

Despite that limestone is the most frequent rock type in climbing routes, there are other rock types in the climbing area including marble or schist. Many authors agree that the impact on plant communities over different rock types could be quite different [10,11]. As such, these studies need to be extended to include different rock types.

In the present study, several variables often measured as indicators or proxies of climbing disturbance such as limb removal, bark abrasion, reductions in average leaf size, or in flower number or size, or differences in growth rate, colony size, or reproductive rate [17] were disregarded because of the sampling design used. That is, we selected transects by pairs (i.e. climbed/not climbed) in order to avoid differences involving the uncontrolled environmental factors (both biotic or abiotic ones). In contrast, climbed and unclimbed areas were sampled separately in most of the studies [10,9], so that the differences between climbed and unclimbed plots could be explained only by a combination of rock morphology, environment, and climbing pressure [10], hindering the evaluation of the impact of climbing activities alone. However, in a second step we might include some environmental key factors (e.g. rock type, soil nutrients, soil moisture, etc.). Evaluating the influence of these environmental factors over plant community enabled us to use them as predictor variables to foresee the impact of climbing activities and to make management decisions accordingly.

### Management guidelines

There is an enormous increase in requests for new climbing routes in protected areas in Spain, especially the Mediterranean part ([49], A.J. Herrera and D. Cuerda pers. com.). However, in many climbing areas, conservation problems have been detected, which urgently need to be addressed based on scientifically sound evidence as demanded by climber associations (e.g. FEDME; www.fedme.es).

**Fig 3:**
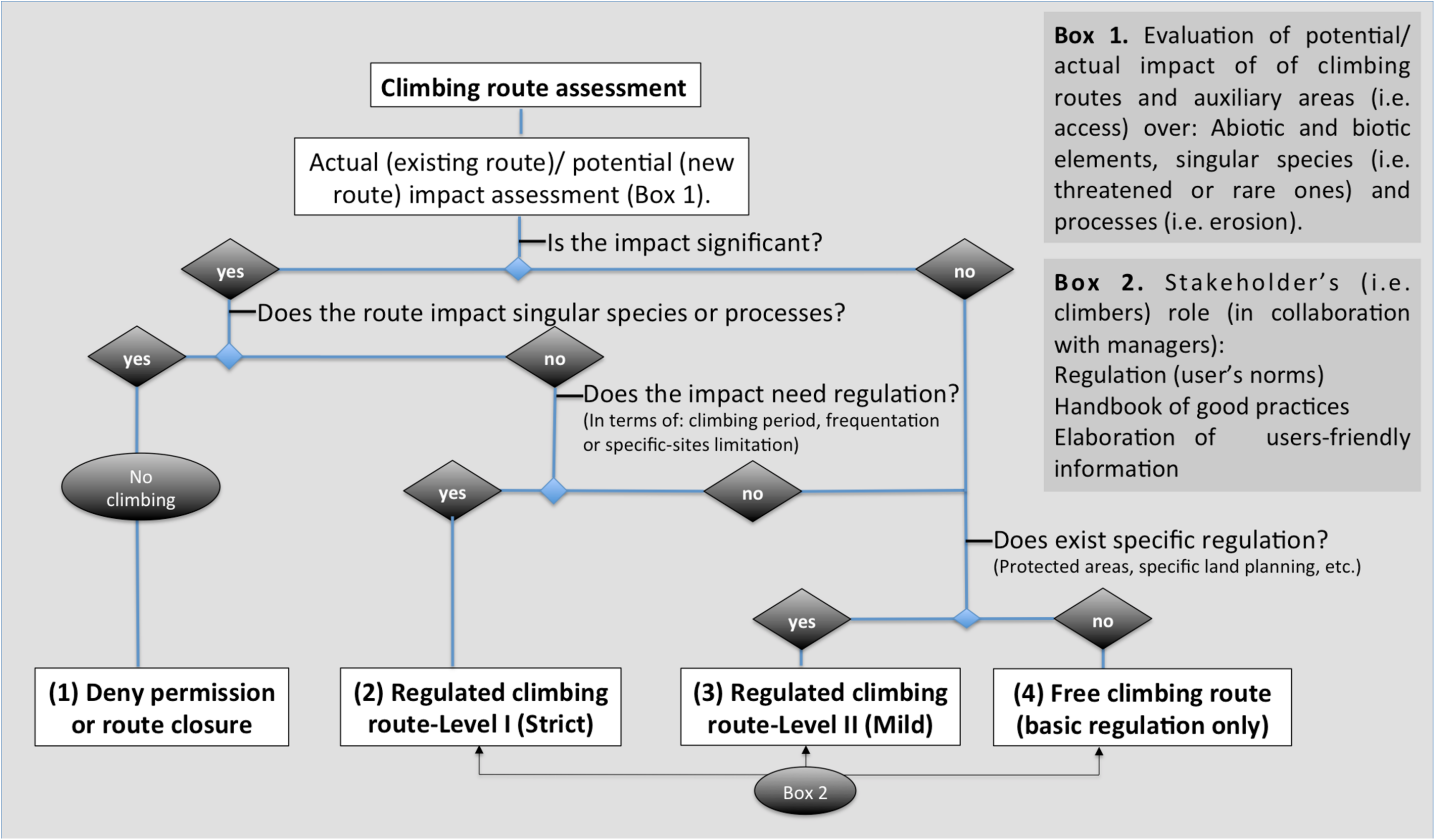
Pathway decision scheme showing the proposed guidelines for the assessment of rock-climbing routes (see text for further details).

We propose a protocol to evaluate climbing routes (Fig 3). All the existing and proposed routes need to be assessed for their impact on biotic and abiotic elements. Also, the impact on endemic/threatened/rare species need be established. Based on these results, managers can decide, following a pathway decision scheme (see Fig 3), whether the impact is significant for species or environmental factors (i.e. key species or soil erosion). Then, if the route affects singular species (i.e. threatened or rare species) or other particular elements (i.e. specific rock types, geological forms, etc.), managers should recommend route closure or deny permission to open new ones (Decision 1). If not, then the following step is to decide whether the impact needs regulation (limiting climbing period, frequentation or specific sites), resulting in a strictly regulated climbing route (Decision 2). Even if the impact does not need regulation, any other specific regulations might apply, as in protected areas or other land-planning measures. For this situation a mildly-regulated climbing route is proposed (Decision 3). Finally, in areas that do not fulfil the former questions (i.e. cliffs in peri-urban areas) climbing intensity or type could be more flexible, becoming “free climbing route” (i.e. climbing routes with high potential for frequent climbing, even the more accessible equipped routes for climbers and walkers called "Via Ferrata")(Decision 4). In regulated climbing routes (strict and mild regulated ones) and free climbing routes stakeholders must play an important role in establishing specific regulations, in proposing and accepting commitment to good practices, and in preparing and disseminating user-friendly information about regulations and good practices. These might be established by means of participation process guided by administration managers.

## Conclusions

On the basis of this study, we conclude that the impact of rock climbing on the plant community (in terms of cover, richness, and composition) is detrimental and that it strongly depends on climbing frequency, being especially worrisome for overcrowded routes. Closure or controlled access to frequently climbed areas would be necessary to prevent species loss and avoid changes in cliff plant communities. However, implementing unpopular measures such as total or partial closure of existing climbing routes, must be supported by solid information and assessment. Managers would need to make an initial assessment of the impact before giving permission for a route. For this reason, studies might relate the effect to several predictor variables that allow managers the impact to be foreseen in a given area. On this basis, managers could make a decision or request more data. This is a particular need in areas with relict, and/or endemic, and/or threatened plants. Therefore, it is necessary to study more sites, with different rock types and different environmental conditions in order to clearly define the patterns of impact and the peculiarities concerning this growing management challenge that is jeopardizing the conservation of this original and fragile Mediterranean habitat.

## Supporting information

S1 Table Results of permanova analysis. Significant values in bold (formula = data_ species ~ Site * Use; permutations = 999). Significant codes for p-values obtained after the GLMMs:. p< 0.1; * p< 0.05; **p< 0.01; ***p< 0.001.
(DOCX)

S2 Table Results of the permutation test performed after the variables of CCA. CCA1-CCA2 = CCA scores of the first two canonical axes. R2 of the model. P-values obtained after permutation test (n permutations = 999) as follows: **p< 0.01; ***p< 0.001.
(DOCX)

S1 File Sampling dataset. (TXT)
