# Supplementary material for: Rock climbing alters plant species composition, cover, and richness in Mediterranean limestone cliffs
Source: PLoS One. 2017 Aug 2;12(8):e0182414. doi: 10.1371/journal.pone.0182414 (PMC5540606; doi:10.1371/journal.pone.0182414)
Supplement: S1 Table — Significant values in bold (formula = data_ species ~ Site * Use; permutations = 999). Significant codes for p-values obtained after the GLMMs:. p< 0.1; * p< 0.05; **p< 0.01; ***p< 0.001. (DOCX) [file pone.0182414.s001.docx]

**S1 Table**.

|  | **Df** | **SumsOfSqs** | **MeanSqs** | **F.Model** | **R^2^** | **Pr(>F)** |
| --- | --- | --- | --- | --- | --- | --- |
| **Site (Cabra/Alfacar/Cahorros)** | 2 | 1.7740 | 0.88702 | 2.7180 | 0.13843 | **0.001**** |
| **Use (climbed/unclimbed)** | 1 | 1.2827 | 1.28274 | 3.9305 | 0.10009 | **0.001**** |
| **Site * Use** | 2 | 0.9476 | 0.47378 | 1.4517 | 0.07394 | **0.044 **** |
| Residuals | 27 | 8.8115 | 0.32635 |  | 0.68755 |  |

Table S2. Results of the permutation test performed after the variables of CCA. CCA1-CCA2= CCA scores of the first two canonical axes. R^2^ of the model. P-values obtained after permutation test (n permutations=999) as follows: **p< 0.01; ***p< 0.001.

| **Variable** | **CCA1** | **CCA2** | **R^2^** | **Pr(>r)** |
| --- | --- | --- | --- | --- |
| Alcandora | -0.33741 | 0.94136 | 0.5618 | **0.000999***** |
| Alfacar | -0.61099 | -0.79164 | 0.4493 | **0.000999***** |
| Cahorros | 0.99798 | 0.06353 | 0.2946 | **0.008991**** |
| climbed | 0.92712 | -0.37476 | 0.3133 | **0.002997**** |
| unclimbed | -0.92712 | 0.37476 | 0.3133 | **0.002997**** |
| cover | -0.86117 | 0.50832 | 0.4394 | **0.000999***** |
| richness | -0.99964 | -0.02665 | 0.6247 | **0.000999***** |
