# Supplementary material for: Rock climbing alters plant species composition, cover, and richness in Mediterranean limestone cliffs
Source: PLoS One. 2017 Aug 2;12(8):e0182414. doi: 10.1371/journal.pone.0182414 (PMC5540606; doi:10.1371/journal.pone.0182414)
Supplement: S2 Table — CCA1-CCA2 = CCA scores of the first two canonical axes. R2 of the model. P-values obtained after permutation test (n permutations = 999) as follows: **p< 0.01; ***p< 0.001. (DOCX) [file pone.0182414.s002.docx]

**S2 Table.**

| **Variable** | **CCA1** | **CCA2** | **R^2^** | **Pr(>r)** |
| --- | --- | --- | --- | --- |
| Cabra | -0.33741 | 0.94136 | 0.5618 | **0.000999***** |
| Alfacar | -0.61099 | -0.79164 | 0.4493 | **0.000999***** |
| Cahorros | 0.99798 | 0.06353 | 0.2946 | **0.008991**** |
| climbed | 0.92712 | -0.37476 | 0.3133 | **0.002997**** |
| unclimbed | -0.92712 | 0.37476 | 0.3133 | **0.002997**** |
| cover | -0.86117 | 0.50832 | 0.4394 | **0.000999***** |
| richness | -0.99964 | -0.02665 | 0.6247 | **0.000999***** |
